# Temperature-Dependent Morphology Modulation of MoO_2_ from 1D Nanoribbons to 2D Nanoflakes for Enhanced Two-Dimensional Electrode Applications

**DOI:** 10.3390/nano15050392

**Published:** 2025-03-04

**Authors:** Di Wu, Tianrong Yi, Yutao Hu, Jianxiong Xie, Yu Deng, Junqi He, Yuting Sun, Jidong Liu, Qiaoyan Hao, Wenjing Zhang

**Affiliations:** 1 State Key Laboratory of Radio Frequency Heterogeneous Integration, Institute of Microscale Optoelectronics, Shenzhen University, Shenzhen 518060, China; physicswudi@hnnu.edu.cn (D.W.); yitianrong2022@email.szu.edu.cn (T.Y.); huyutao2022@email.szu.edu.cn (Y.H.); jzxx6844@163.com (J.X.); dengyu2022@email.szu.edu.cn (Y.D.); hjq20010610@163.com (J.H.); m13327511015_3@163.com (Y.S.); ljd@szu.edu.cn (J.L.); 2 College of Electronic Engineering, Huainan Normal University, Huainan 232038, China

**Keywords:** MoO_2_, morphology modulation, chemical vapor deposition, two-dimensional electrode

## Abstract

The morphology modulation of target crystals is important for understanding their growth mechanisms and potential applications. Herein, we report a convenient method for modulating the morphology of MoO_2_ by controlling different growth temperatures. With an increase in growth temperature, the morphology of MoO_2_ changes from a nanoribbon to a nanoflake. Various characterization methods, including optical microscopy, atomic force microscopy, (vertical and tilted) scanning electron microscopy, Raman spectroscopy, high-resolution transmission electron microscopy, and selected area electron diffraction, were performed to unveil the morphology modulation and lattice structure of MoO_2_. Both MoO_2_ nanoribbons and nanoflakes display a standing-up growth mode on c-sapphire substrates, and their basal planes are MoO_2_(100). Further investigations into devices based on MoS_2_ with Au/Ti/MoO_2_ electrodes show the potential applications of MoO_2_ in two-dimensional electrodes. These findings are helpful for the synthesis of MoO_2_ with different morphologies and applications in the field of optoelectronic nanodevices.

## 1. Introduction

Molybdenum oxides have been extensively studied due to their distinct physicochemical properties and wide range of applications in photodetectors, photocatalysts, electronics, and organic photovoltaic devices [1,2,3,4,5]. Among them, molybdenum trioxide (MoO_3_) and molybdenum dioxide (MoO_2_) have attracted much more research interest [6,7]. While MoO_3_ exhibits the features of an n-type semiconductor with a large bandgap (~3.2 eV) [5,7], MoO_2_ exhibits the features of a metal with a monoclinic structure and lattice parameters of *a* = 5.62 Å, *b* = 4.86 Å, *c* = 5.63 Å, and *β* = 120.93° (ICSD-152316) and possesses a high melting point and excellent chemical stability [8,9,10]. The high electronic conductivity of MoO_2_ indicates its potential as a promising anode material for next-generation lithium-ion batteries [11]. Its low-symmetry structure endows MoO_2_ with highly anisotropic electrical and optical properties [12,13]. In addition, MoO_2_ nanosheets show linear magnetoresistance [9,14], showing potential for applications in new magnetic devices.

Due to its non-layered structure, the morphology of MoO_2_ can be modulated by controlling different growth conditions, such as substrate and precursor density [6,8,12,15,16]. In fact, different morphologies of MoO_2_ have been achieved over the past decades. For instance, 1D MoO_2_ nanorods were prepared via a thermal decomposition method, showing great capacitive behavior [17]. Chemical vapor deposition (CVD) is one of the most popular and controllable synthesis methods for producing high-quality 2D materials. Various factors, including precursors, gas flow, substrate, and growth temperature (GT), can influence the structure, composition, and morphology of target crystals [18,19,20,21]. MoO_2_ nanosheets on SiO_2_/Si substrates synthesized via the CVD method exhibited great in-plane anisotropy at an electrical conductivity ratio of about 10.1, which demonstrated potential for application in multifunctional integrated plasmonics and ion-inspired electronics devices [12]. Recently, MoO_2_ nanoflakes were investigated in the lying-down and standing-up growth modes on c-sapphire substrates, and the products showed morphological competition during the growth process [8]. Consequently, the morphology modulation of MoO_2_ is essential for understanding their growth mechanism, as well as exploring their applications in different areas.

In this article, MoO_2_ was synthesized through the atmospheric-pressure chemical vapor deposition (APCVD) method, using MoO_3_ powder and H_2_ as the precursor and reductant, respectively. The effects of GT on MoO_2_ morphology were systematically investigated. The morphology of as-prepared MoO_2_ crystals changed from a nanoribbon to a nanoflake with an increase in GT. Comprehensive characterization techniques, including optical microscopy (OM), atomic force microscopy (AFM), scanning electron microscopy (SEM), Raman spectroscopy, high-resolution transmission electron microscopy (HRTEM), and selected area electron diffraction (SAED), were performed to elucidate the morphology modulation and lattice structure of MoO_2_. The basal planes of both MoO_2_ nanoribbons and nanoflakes were unveiled to be MoO_2_(100), and the growth direction of MoO_2_ nanoribbons was MoO_2_<010>. Energy-dispersive X-ray spectrometry (EDX) measurements showed a stoichiometric ratio of ~2.11 between the Mo and O elements in an as-prepared sample, a result that was consistent with the composition of MoO_2_. Due to its high electronic conductivity, MoO_2_ can be used as an electrode material for nanodevices. Field-effect transistor (FET) devices based on MoS_2_ nanoflakes were then fabricated with Au/Ti and Au/Ti/MoO_2_ electrodes, showing better performance for Au/Ti/MoO_2_ electrodes. These findings offer valuable insights into the synthesis of MoO_2_ with different morphologies and show their potential for application in two-dimensional (2D) electrode and optoelectronic nanodevices.

## 2. Materials and Methods

### 2.1. Sample Synthesis Method

The MoO_2_ crystals were prepared in an APCVD system, as shown in Supplementary Figure S1a, with one heating zone. MoO_3_ powder (99.95%, Aladdin, Shanghai, China) was treated as the metal precursor, and hydrogen as the reductant. Before conducting the growth experiments, acetone, isopropanol, and DI water were used to preclean the c-sapphire substrates. During the growth process, the c-sapphire substrates were supported with a SiO_2_/Si sheet and placed above the metal precursor within a quartz boat, which was placed in the center of the heating zone. The APCVD system was initially heated from room temperature to 150 °C, with an argon flow rate of 200 sccm. After holding the temperature at 150 °C for 10 min, the APCVD system was heated to GT for 40 min. When the temperature reached GT, hydrogen was introduced at 0.5 sccm, and the argon flow changed to 40 sccm. The growth process lasted for 20 min. After the growth process ended, the system temperature was dropped to 600 °C, followed by rapid cooling at an argon flow of 200 sccm. The temperature–time profile for the growth experiment is shown in Supplementary Figure S1b.

### 2.2. Device Fabrication

The MoS_2_ nanoflakes were transferred onto SiO_2_/Si substrates via the PMMA-assisted method, and the MoO_2_ nanoflakes were transferred onto MoS_2_ nanoflakes via the PDMS-assisted method, as shown in Supplementary Figure S2. The photoresist (AZ 5214-E, AZ Electronic Materials, Shizuoka, Japan) was coated onto a MoO_2_/MoS_2_ heterostructure at a 4000 rpm spin-coating rate for 60 s and then baked at 105 °C for 1 min. The electrode patterns were exposed using a laser writer (MicroWriter ML 3, Durham Magneto Optics Ltd., London, UK). The exposed part of the photoresist was removed via AZ726 MIF (AZ Electronic Materials, Shizuoka, Japan) for 30 s. Additionally, the developer was removed by floating the photoresist in DI water for 30 s. Finally, 10 nm/50 nm Ti/Au metals were deposited onto the samples to fabricate the electrodes through e-beam deposition.

### 2.3. Sample Characterization

The OM measurements were performed via Leica optical microscopy (Leica DM2700M RL, Wetzlar, Germany). The Raman spectra were selected using a Raman microscope (WITec alpha 300R, Lise-Meitner-Str., 6 D-89081 Ulm, Germany) under a 532 nm laser with 600 lines/mm grating. The SEM measurements were carried out using a Zeiss Sigma HD scanning electron microscope (Carl Zeiss AG, Oberkochen, Germany). The AFM measurements were performed under ambient conditions via a Bruker Dimension Icon AFM (365 Boston Rd., Billerica, MA, USA). The TEM, EDX, and SAED measurements were conducted in a JEM-3200FS transmission electron microscope (JEM-3200FS, JEOL, Street No. 6, Haidian District, Beijing, China) using an acceleration voltage of 300 kV.

## 3. Results and Discussion

**Figure 1:**
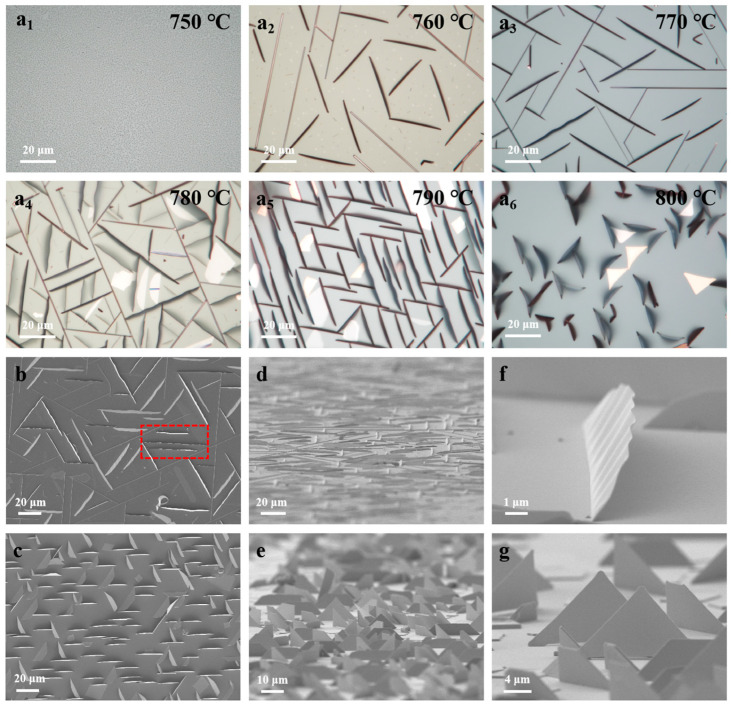
(**a_1_**–**a_6_**) OM images of MoO_2_ on c-sapphire substrates grown at different GTs. (**b**,**c**) Vertical, (**d**,**e**) tilted, and (**f**,**g**) close-up SEM images of MoO_2_ nanoribbons and nanoflakes, respectively.

Figure 1(a_1_–a_6_) displays OM images of MoO_2_ on c-sapphire substrates grown at different GTs. When the GT is 750 °C, only particles can be observed on the substrate, indicating that MoO_2_ nanoribbons and nanoflakes cannot be achieved at a growth temperature of 750 °C. After raising the GT by 10 °C, MoO_2_ nanoribbons tens of micrometers in length were achieved on the substrate. Although these samples looked like nanorods [6], they exhibited a standing-up growth mode. As a result, correctly evaluating the width of the MoO_2_ nanoribbons was difficult. With a continuous increase in GT, the length of the MoO_2_ nanoribbons became shorter. Furthermore, the products displayed an obvious defocus, indicating that MoO_2_ grew along the out-plane direction. When the GT was 800 °C, standing-up MoO_2_ nanoflakes predominantly grew on the substrate. To study the modulation of GT on the morphology of MoO_2_, MoO_2_ samples grown at different temperatures were transferred onto silicon substrates. As shown in Supplementary Figure S3, it was observed that, by raising the GT to 800 °C, MoO_2_ transformed from a 1D nanoribbon to a 2D nanoflake gradually. In fact, due to the standing-up growth mode, the width of the MoO_2_ exhibited significant changes even though its morphology transformed from that of a 1D nanoribbon to a 2D nanoflake. In addition to the standing-up samples, a few lying-down MoO_2_ nanoflakes in the shapes of triangles and isosceles trapezoids were observed on the substrate, which may be due to the inevitable vibration during the sample transfer process.

To unveil the growth behavior of MoO_2_ with different morphologies, SEM measurements were further performed. Figure 1b,c show the SEM images taken along the out-plane direction. As shown in Figure 1b, MoO_2_ grown at lower GTs clearly exhibited a nanoribbon morphology. The unique wavy texture along the growth direction suggests that the grown MoO_2_ nanoribbons are flexible. It is worth noting that a shaded area can be observed on one side of the MoO_2_ nanoribbon (marked by a dashed red rectangle), indicating the standing-up growth mode of the nanoribbons. At higher GTs, the grown MoO_2_ nanoflakes displayed shorter lengths and wider widths, as shown in Figure 1c. Consistent with the nanoribbon sample, a shaded area also appeared on one side of the MoO_2_ nanoflakes due to the standing-up growth mode. Figure 1d,e present the SEM images of MoO_2_ nanoribbons and nanoflakes taken at rotation angles of 30°, respectively, which clearly demonstrates the standing-up growth mode of MoO_2_. The defocus phenomenon in the rotated SEM images is due to over-focusing and under-focusing during rotation-angle induction. A close-up SEM image of the MoO_2_ nanoribbon, as shown in Figure 1f, indicates that the basal plane of the nanoribbon formed an included angle with the substrate surface, resulting in the presence of the shaded area in Figure 1b. A wavy structure was seen on the top side of the MoO_2_ nanoribbon, which may be due to the residual strain induced by lattice mismatch between the MoO_2_ and substrate. The MoO_2_ nanoflakes also displayed a standing-up growth mode, and their morphologies were in the shapes of triangles and right-angled trapezoids, as shown in Figure 1g. In fact, the MoO_2_ nanoflakes exhibited the shape of right-angled trapezoids instead of isosceles trapezoids, arising from the rotation of the MoO_2_ sample. The above results clearly prove the existence of the standing-up growth mode and morphology change from a 1D nanoribbon to a 2D nanoflake of MoO_2_ under different GTs.

**Figure 2:**
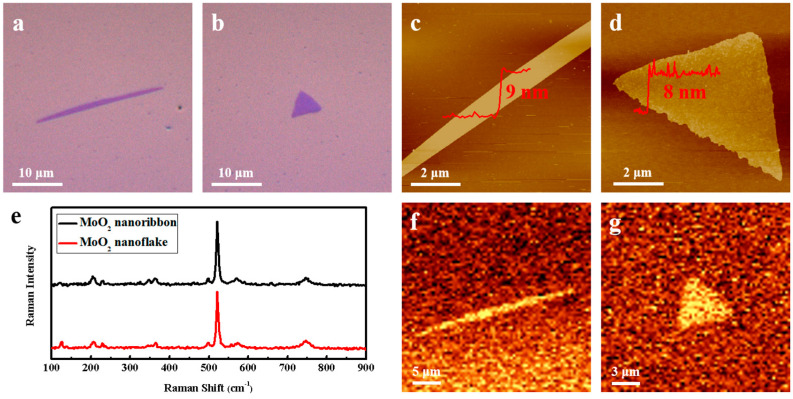
(**a**,**b**) OM and (**c**,**d**) AFM images of a MoO_2_ nanoribbon and a nanoflake on SiO_2_/Si substrates. (**e**) Raman spectra and (**f**,**g**) Raman mapping images of the MoO_2_ nanoribbon and nanoflake, respectively.

Figure 2a,b display OM images of MoO_2_ transferred onto SiO_2_/Si substrates grown at different GTs, showing the morphology transformation from a 1D nanoribbon to a 2D nanoflake. Figure 2c,d present corresponding AFM images of the MoO_2_ nanoribbon and nanoflake, in which the thicknesses were determined to be 9 and 8 nm, respectively, according to their height profiles. The Raman spectrum of the MoO_2_ nanoribbon (nanoflake), as shown in Figure 2e and Supplementary Table S1, displays Raman peaks at 121.6 (126.4), 205.0 (207.4), 228.7 (228.7), 348.4 (346.1), 362.4 (364.7), 498.7 (498.7), 571.7 (573.9), and 744.9 (747.1) cm^−^^1^, results that are in agreement with those of previous reports [5,9,14]. Figure 2f shows the Raman mapping images of the MoO_2_ nanoribbon and nanoflake at 745 cm^−^^1^, indicating the uniform quality of these samples. In addition, Supplementary Figure S4a exhibits the XRD patterns of the MoO_2_ nanoribbon and nanoflake, suggesting the formation of high-quality MoO_2_ phase. Supplementary Figure S4b presents the XPS spectra of the MoO_2_ nanoribbon and nanoflake. According to the Mo 3*d* core-level emission, the two major peaks located at around 232.3 (Mo 3*d*_3/2_) and 229.2 eV (Mo 3*d*_5/2_) indicate the presence of Mo (+4). The XPS results are consistent with the values for MoO_2_ reported previously [10].

**Figure 3:**
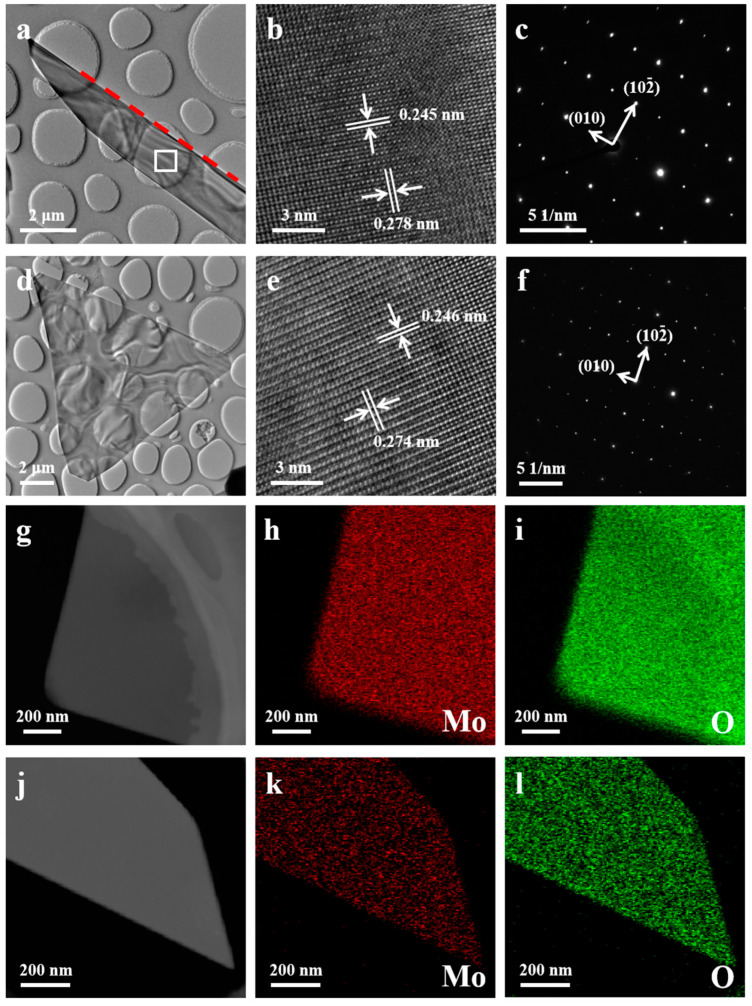
The LMTEM images, HRTEM images, and SAED patterns of (**a**–**c**) a MoO_2_ nanoribbon and (**d**–**f**) a MoO_2_ nanoflake, respectively. A TEM image and elementary mapping images of the Mo and O elements of the MoO_2_ (**g**–**i**) nanoribbon and (**j**–**l**) nanoflake, respectively.

To investigate the microstructure of the as-grown MoO_2_ samples, TEM and SAED measurements were performed, as shown in Figure 3. The MoO_2_ nanoribbon and nanoflake were transferred onto a holey carbon grid through a simple immersion and scraping method. Figure 3a displays a low-magnification TEM image of the MoO_2_ nanoribbon. The red dashed line in Figure 3a represents the side of the MoO_2_ nanoribbon adjacent to the substrate. The HRTEM image, as shown in Figure 3b, was selected in the white rectangle area in Figure 3a. The spacing between the two planes was determined to be 0.245 and 0.278 nm, corresponding to the (020) and (10−2) planes of MoO_2_, respectively [6,14]. Figure 3c presents the corresponding SAED pattern of the MoO_2_ nanoribbon. The real distances between two spaces, ~0.483 and 0.276 nm, were calculated based on the diffraction spots, corresponding to the (010) and (10−2) planes of MoO_2_, respectively [6,14]. A low-magnification (LM) TEM image of the MoO_2_ nanoflake is presented in Figure 3d, showing a similar contour to that in the OM results. Figure 3e,f display the HRTEM and SAED results of the MoO_2_ nanoflake, similar to that of the MoO_2_ nanoribbon, indicating that controlling GT only modulates the morphology of the target MoO_2_, rather than the crystal lattice. Figure 3g shows the dark-field TEM image of an individual MoO_2_ nanoflake. The corresponding EDX mapping images are presented in Figure 3h,i, showing uniform chemical composition in MoO_2_. Figure 3j–l present the corresponding results for the MoO_2_ nanoribbon. Supplementary Figure S5 displays the EDX spectrum of the as-prepared sample, showing a 1:2.11 stoichiometric ratio between the Mo and O elements, confirming that the as-grown products were MoO_2_.

**Figure 4:**
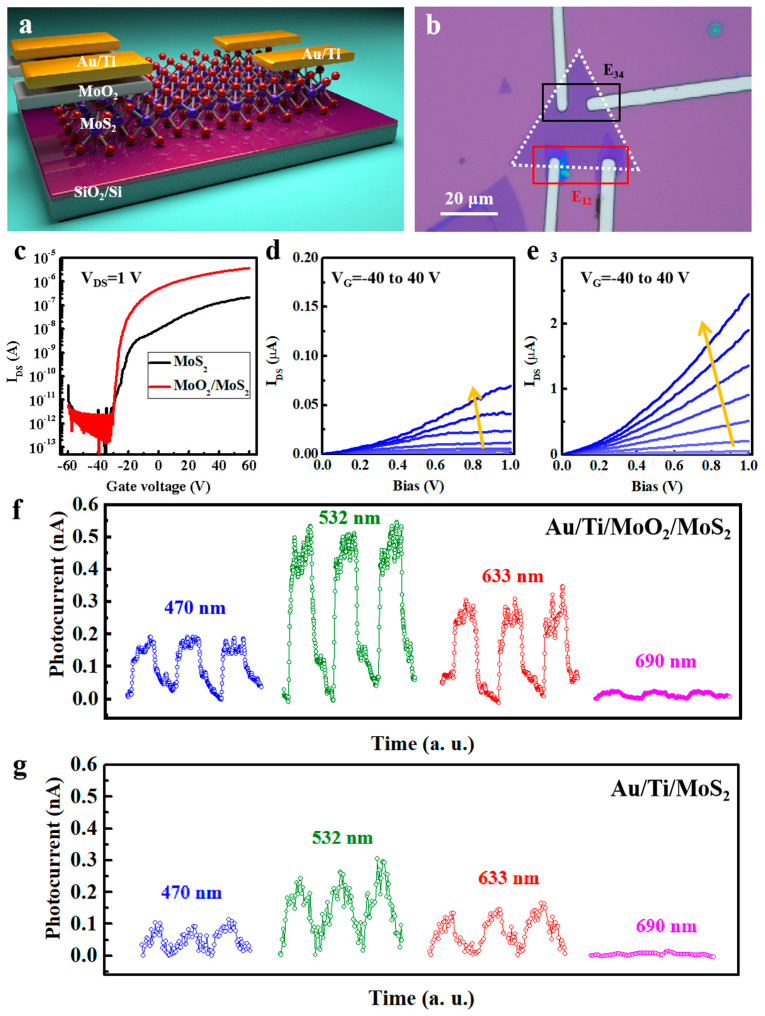
(**a**) A diagram of the MoS_2_ FET device fabricated with Au/Ti/MoO_2_ and Au/Ti electrodes. (**b**) An OM image of the MoS_2_ FET device with Au/Ti and Au/Ti/MoO_2_ electrodes. (**c**) The transfer curves of the MoS_2_ FET device with Au/Ti and Au/Ti/MoO_2_ electrodes. The output curves of the MoS_2_ FET device with (**d**) Au/Ti and (**e**) Au/Ti/MoO_2_ electrodes. The time-dependent photocurrent curve of the MoS_2_ FET device with (**f**) Au/Ti/MoO_2_ and (**g**) Au/Ti electrodes under 470, 532, 633, and 690 nm laser irradiation.

According to the *I*–*V* curves of the as-prepared MoO_2_ nanoribbon and nanoflake in Supplementary Figure S6a,b, their electric conductivities were 5.6 × 10^4^ and 2.2 × 10^4^ S/cm at room temperature, respectively. Supplementary Table S2 lists the performance comparison among the MoO_2_ nanoribbon and nanoflake with previous reports, confirming the high electric conductivity of the as-synthesized samples. The MoO_2_ nanoflake is able to sustain stability until it approaches its electrical breakdown point at a V_DS_ of 6 V and a J_DS_ of 6.4 × 10^7^ A/cm^2^, as shown in Supplementary Figure S6c. Due to the atomic thickness of 2D materials, such as single-layered MoS_2_, the Fermi-level pinning effect is present in the interfaces between 2D materials and metal electrodes, arising from several factors, such as the defects created by the high-energy metal deposition and lithography process [22]. Therefore, MoO_2_ nanoflakes can be employed as a 2D electrode material for devices based on ultrathin 2D materials to reduce the surface states from defects or residues at the contact point between metals and semiconductors. The AFM image of device and the typical Raman spectra of MoS_2_ and MoO_2_/MoS_2_ are displayed in Supplementary Figure S7, indicating that the MoS_2_ domain on the substrate was monolayer [23,24,25]. The as-prepared MoO_2_ nanoflakes are transferred onto a monolayer MoS_2_ to protect the MoS_2_. Figure 4a,b show a schematic diagram and an OM image of the fabricated device, respectively. One pair of electrodes (electrodes 1 and 2, E_12_) was deposited on the MoO_2_/MoS_2_ area as drain and source electrodes. For comparison, another pair of electrodes (electrodes 3 and 4, E_34_) was directly deposited on an individual MoS_2_ domain. Figure 4c presents the transfer curves of a MoS_2_ device fabricated with E_12_ and E_34_ electrodes, showing the n-type transport feature [26,27,28]. The source–drain current of the MoS_2_ channel with an E_12_ electrode was one order of magnitude higher than that with an E_34_ electrode under *V_G_* = 40 V, suggesting better interface condition between MoO_2_ and MoS_2_. The electron mobility (*μ*) of the device was calculated from the transfer curves according to the following equation:

(1) $$\mu =\frac{L}{W}\frac{d}{\varepsilon_{0}\varepsilon_{r}}\frac{1}{V_{DS}}\frac{dI_{DS}}{dV_{G}},$$

where *L* and *W* are the channel length and width of the device, *ε*_0_ = 8.85 × 10^−12^ F·m^−1^ is the vacuum permittivity, *ε_r_* = 3.9 is the relative permittivity of the SiO_2_ dielectric layer, and *d* = 300 nm is the thickness of the SiO_2_ layer. The electron mobility of the MoS_2_ device is 9.04 cm^2^/V·s with Au/Ti/MoO_2_ electrodes and 0.284 cm^2^/V·s with Au electrodes. Figure 4d,e display the output curves of the MoS_2_ device fabricated with E_12_ and E_34_ electrodes under different *V_G_* values, respectively, further suggesting better electrical performance with the Au/Ti/MoO_2_ electrodes. The yellow arrows in Figure 4d,e represent the increasing trend of *V_G_* value. Figure 4f,g present the time-dependent photocurrent measurements of a photodetector based on MoS_2_ with Au/Ti/MoO_2_ and Au/Ti electrodes and illuminated using 470, 532, 633, and 690 nm lasers. To evaluate the photoresponse performance, the device’s figures of merit, including photoresponsivity (*R*), specific detectivity (D*), and external quantum efficiency (*EQE*), were calculated by the following equations:

(2) $$R=\frac{I_{ph}}{PS},$$

(3) $$D^{*}=R\sqrt{\frac{S}{2qI_{dark}}},$$

(4) $$EQE=\frac{R}{\left(hc/e\lambda \right)},$$

where *P* is the power density, *S* is the effective area of the device, *h* is the Planck constant, and *c* is the speed of light [29,30,31]. It should be noted that the effective area, *S*, was defined as the entire MoS_2_ due to the fact that it was not patterned. Therefore, the calculated photoresponse performance was conservative and lower than the actual value according to the equations. Supplementary Figure S8 plots the evolution of the above figures of merit under different power densities at 532 nm laser illumination. In particular, the device fabricated with Au/Ti/MoO_2_ electrodes exhibited a maximum *R* value of 9.0 A·W^−1^ at a low light power density of 0.05 mW·cm^−2^. Meanwhile, the device exhibited the highest D* and *EQE* values of 3.5 × 10^11^ Jones and 2028%, respectively. For the sake of comparison, Supplementary Figure S9 compares the photoresponse performance between the MoS_2_ devices fabricated with and without the MoO_2_ layer, demonstrating more efficient photoresponse for the MoS_2_ device with Au/Ti/MoO_2_ electrode. These experimental results indicate that the introduction of MoO_2_ between a metal electrode and MoS_2_ can effectively protect MoS_2_ from degeneration and improve the device’s performance.

## 4. Conclusions

In summary, MoO_2_ nanoribbons and nanoflakes were grown following the APCVD method and characterized via OM, AFM, Raman spectroscopy, SEM, and TEM. The morphology of MoO_2_ was modulated from a nanoribbon to a nanoflake by increasing the growth temperature. Such MoO_2_ samples exhibited a standing-up growth mode instead of a lying-down one, and both their basal planes were MoO_2_(100). The FET device based on Au/Ti/MoO_2_ electrodes showed better electrical performance due to the protection of MoS_2_ from the deposited metal electrodes. Our findings provide a convenient method for synthesizing MoO_2_ and modulating its morphology. The target MoO_2_ may have great potential to be used as an electrode for novel 2D material-based optoelectronic devices.

## Data Availability

The data are contained within this article and its Supplementary Materials.

## Supplementary Materials

The following supporting information can be downloaded at https://www.mdpi.com/article/10.3390/nano15050392/s1. Figure S1: The APCVD system and growth condition of MoO_2_. Figure S2: A schematic of the transfer process of MoO_2_. Figure S3: The morphology evolution of MoO_2_. Figure S4: The XRD patterns and XPS spectra of the MoO_2_ nanoribbons and nanoflakes. Figure S5: The EDX spectrum of MoO_2_. Figure S6: The electrical measurements of MoO_2_. Figure S7: The AFM image and Raman spectra of MoS_2_ and MoO_2_/MoS_2_ domains. Figure S8: The photoresponse performance of the MoS_2_ device with Au/Ti/MoO_2_ electrodes. Figure S9: The photoresponse performance comparison between the MoS_2_ device with and without the MoO_2_ layer. Table S1: Raman frequencies (cm^−1^) for MoO_2_. Table S2: Summary of several MoO_2_ nanomaterials. Refs. [9,11,14,32,33,34,35,36] are cited in the supplementary materials.
