# Supplementary material for: Temperature-Dependent Morphology Modulation of MoO2 from 1D Nanoribbons to 2D Nanoflakes for Enhanced Two-Dimensional Electrode Applications
Source: Nanomaterials (Basel). 2025 Mar 4;15(5):392. doi: 10.3390/nano15050392 (PMC11901888; doi:10.3390/nano15050392)
Supplement: Supplementary file 1 [file nanomaterials-15-00392-s001.zip › nanomaterials-3473334-supplementary.pdf]

Support Information

Temperature-Dependent Morphology Modulation of MoO_2 from 1D Nanoribbons to 2D Nanoflakes for Enhanced Two-Dimensional Electrode Applications

Di Wu [1,2,†], Tianrong Yi [1,†], Yutao Hu [1], Jianxiong Xie [1], Yu Deng [1], Junqi He [1], Yuting Sun [1], Jidong Liu [1], Qiaoyan Hao [1,*] and Wenjing Zhang [1,*]

[1] State Key Laboratory of Radio Frequency Heterogeneous Integration, Institute of Microscale Optoelectronics, Shenzhen University, Shenzhen 518060, China; physicswudi@hnnu.edu.cn (D.W.); yitianrong2022@email.szu.edu.cn (T.Y.); huyutao2022@email.szu.edu.cn (Y.H.); jzxx6844@163.com (J.X.); dengyu2022@email.szu.edu.cn (Y.D.); hjq20010610@163.com (J.H.); m13327511015_3@163.com (Y.S.); ljd@szu.edu.cn (J.L.)

[2] College of Electronic Engineering, Huainan Normal University, Huainan 232038, China

* Correspondence: hqy@szu.edu.cn (Q.H.); wjzhang@szu.edu.cn (W.Z.)

† These authors contributed equally to this work.

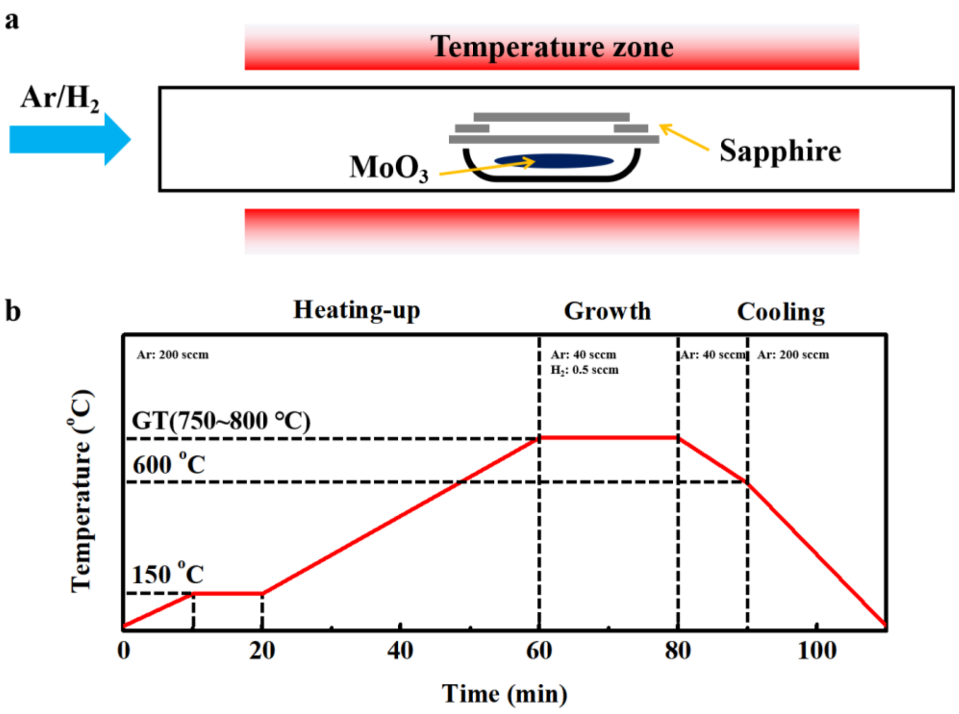

Figure S1. (a) The schematic of the setup of the APCVD system for the growth of MoO_2 nanoribbons and nanoflakes. (b) The temperature-time profile for MoO_2 growth.

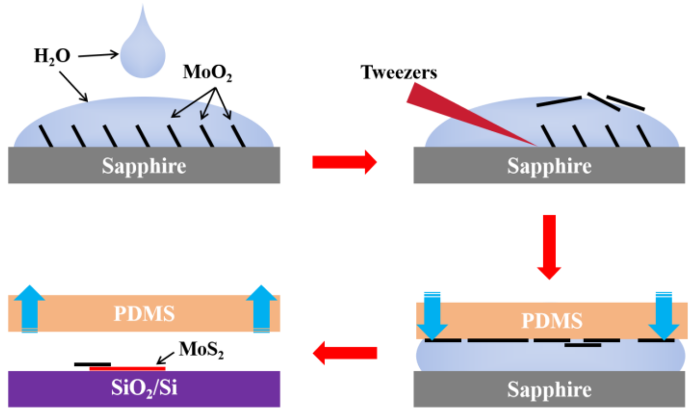

Figure S2. The schematic of transfer process of MoO_2 from sapphire substrate onto SiO_2/Si substrate.

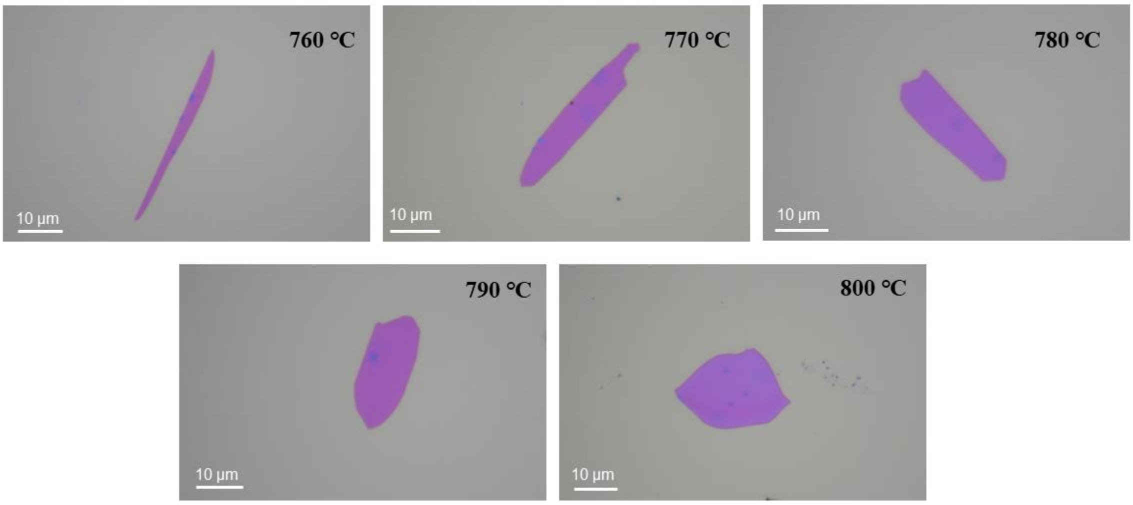

Figure S3. The morphology evolution of MoO_2 from 1D nanoribbon to 2D nanoflake modulated by the growth temperature.

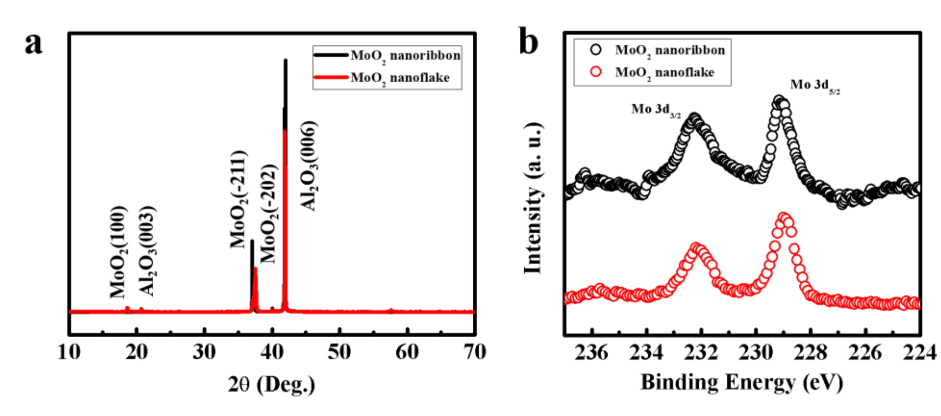

Figure S4. (a) XRD patterns and (b) XPS spectra of MoO_2 nanoribbon and nanoflake.

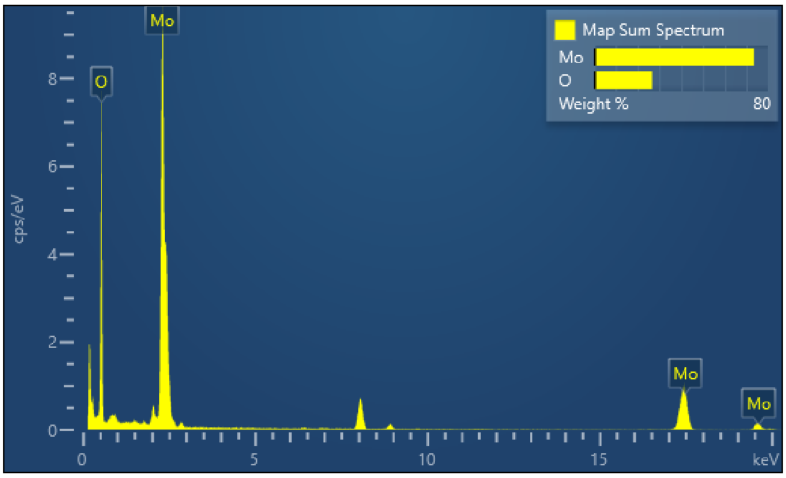

Figure S5. The EDX spectrum of MoO_2.

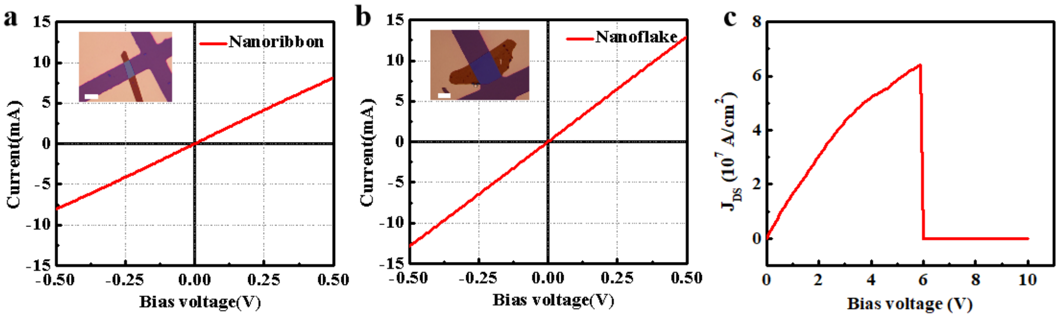

Figure S6. The *I-V* curves of MoO_2 (a) nanoribbon and (b) nanoflake, respectively. The insets are the OM images of devices, scale bar: 10 μm. (c) Breakdown current density of MoO_2 nanoflake.

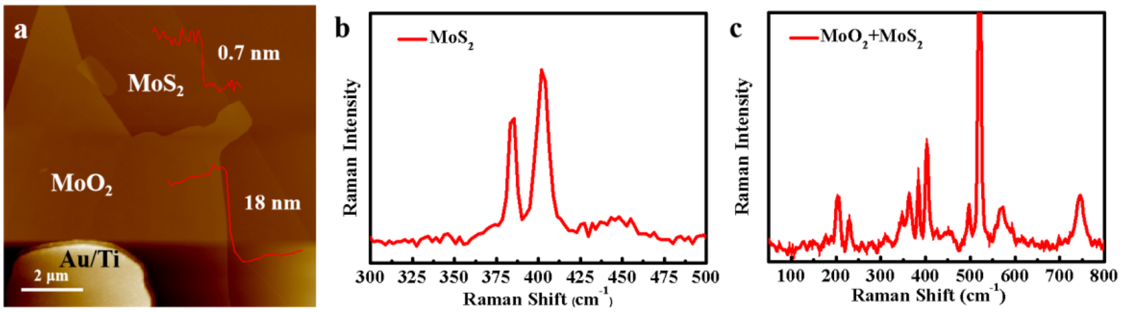

Figure S7. (a) The AFM image of the device. The Raman spectra of (b) MoS_2 and (c) MoO_2/MoS_2 domain, respectively.

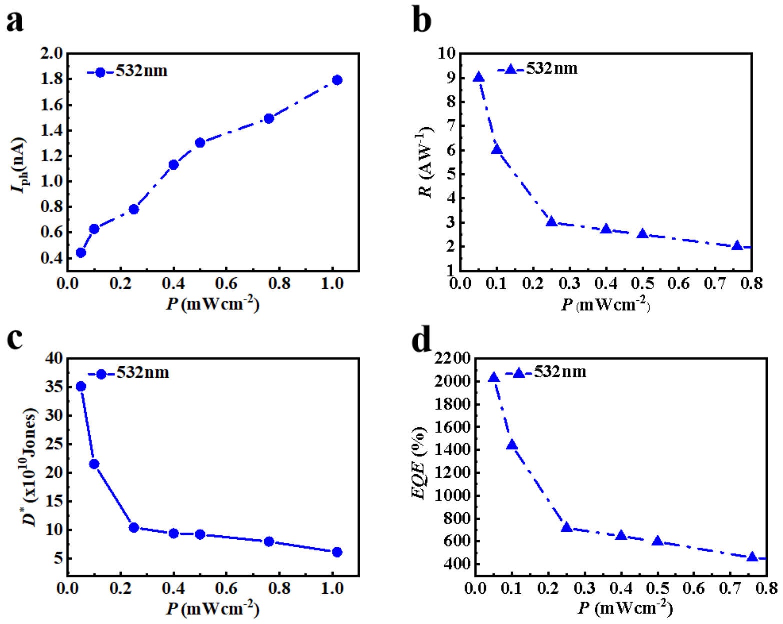

Figure S8. Calculated (a) I_{ph}, (b) R, (c) D^*, and (d) EQE as a function of light power density for the MoS_2 device fabricated with Au/Ti/MoO_2 electrodes.

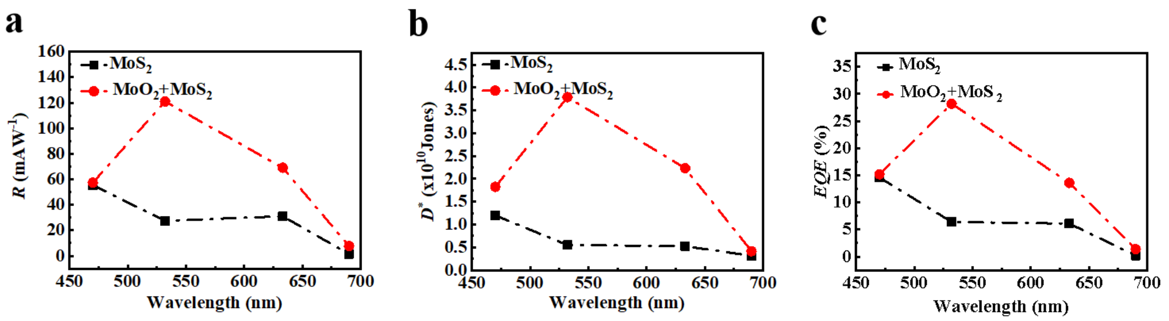

Figure S9. Calculated (a) R, (b) D^*, and (c) EQE as a function of wavelength for the MoS_2 devices fabricated with Au/Ti/MoO_2 and Au/Ti electrodes, respectively. The measurements were performed at a fixed light power density of 0.51 mW·cm^{-2} under ambient conditions.

Table S1. Raman frequencies (cm^{-1}) for the MoO_2 nanoribbon and nanoflake.

Nanoribbon	121.6	205.0	228.7	348.4	362.4	498.7	571.7	744.9
Nanoflake	126.4	207.4	228.7	346.1	364.7	498.7	573.9	747.1
Assignment	A_g $\delta(O_2Mo_2)_n$	A_g $\delta(OMo_2)$	A_g $\delta(OMo_2)$	A_g $\delta(OMo_3)$	A_g $\delta(O{=}Mo)$	B_{1g} $\nu(OMo_3)$	B_{1g} $\nu(OMo_3)$	B_{3g} $\nu(OMo_3)$

Table S2. Summary of the synthesis method and conductivity of low-dimensional MoO_2 materials.

Materials	Synthesis Method	Morphology	Conductivity(S/cm)	Refs.
MoO_2	CVD	nanorods	6.04×10^3	[1]
MoO_2	PLD	films	$>1.40\times10^3$	[2]
MoO_2	CVD	nanorods	59	[3]
MoO_2	CVD	nanoplates	2.50×10^4	[4]
MoO_2	nanocasting synthesis	mesoporous	100	[5]
MoO_2	CVD	nanoflakes	3.91×10^4	[6]
MoO_2	hydrothermal synthesis	nanorods	190	[7]
MoO_2	CVD	nanosheets	200-475	[8]
MoO_2	CVD	nanoflakes	2.20×10^4	this work
MoO_2	CVD	nanoribbons	5.60×10^4	this work

References:

(1) Xie, Q.; Zheng, X.; Wu, D.; Chen, X.; Shi, J.; Han, X.; Zhang, X.; Peng, G.; Gao, Y.; Huang, H., High electrical conductivity of individual epitaxially grown MoO_2 nanorods. *Appl. Phys. Lett.* **2017**, *111*, 093505.

(2) Ma, C. H.; Lin, J. C.; Liu, H. J.; Do, T. H.; Zhu, Y. M.; Ha, T. D.; Zhan, Q.; Juang, J. Y.; He, Q.; Arenholz, E.; Chiu, P. W.; Chu, Y. H., Van der Waals epitaxy of functional MoO_2 film on mica for flexible electronics. *Appl. Phys. Lett.* **2016**, *108*, 253104.

(3) Liu, J.; Shi, J.; Wu, D.; Zheng, X.; Chen, F.; Xiao, J.; Li, Y.; Song, F.; Gao, Y.; Huang, H., Epitaxial growth of <010>-oriented MoO_2 nanorods on m-sapphire. *Curr. Appl. Phys.* **2020**, *20*, 1130-1135.

(4) Zhang, H.; Wu, Y.; Huang, Z.; Shen, X.; Li, B.; Zhang, Z.; Wu, R.; Wang, D.; Yi, C.; He, K.; Zhou, Y.; Liu, J.;Li, B.; Duan, X., Synthesis of Two-Dimensional MoO_2 Nanoplates with Large Linear Magnetoresistance and Nonlinear Hall Effect. *Nano Lett.* **2023**, *23,* 2179-2186.

(5) Shi, Y.; Guo, B.; Corr, S. A.; Shi, Q.; Hu, Y.-S.; Heier, K. R.; Chen, L.; Seshadri, R.; Stucky, G. D., Ordered Mesoporous Metallic MoO_2 Materials with Highly Reversible Lithium Storage Capacity. *Nano Lett.* **2009**, *9*, 4215-4220.

(6) Wu, D.; Li, B.; Wang, Z.; Yuan, L.; Ou, H.; Li, Z.; Yi, T.; Wang, Y.; Liu, J.; Hao, Q.; Weng, X.; Zeng, Y.-J.; Huang, H.; Ouyang, F.; Zhang, W., Unidirectional growth of molybdenum dioxide nanoflakes on C-sapphire substrate via buffer layer induction. *Mater. Character.* **2024**, *216*, 114307.

(7) Hu, B.; Mai, L.; Chen, W.; Yang, F., From MoO_3 Nanobelts to MoO_2 Nanorods: Structure Transformation and Electrical Transport. *ACS Nano.* **2009**, *3*, 478-482.

(8) Pu, E.; Liu, D.; Ren, P.; Zhou, W.; Tang, D.; Xiang, B.; Wang, Y.; Miao, J., Ultrathin MoO_2 nanosheets with good thermal stability and high conductivity. *AIP Advances.* **2017**, *7*, 025015.
